# Improved interfacial-bonding strength and electronic structure properties of graphene-reinforced Al composites by Cu coating

**DOI:** 10.1039/d6ra04391g

**Published:** 2026-10-28

**Authors:** Sin-Hyok Jon, Jae-Bok Ri, Chol Jong, Un-Gi Jong, Songchol Hong

**Affiliations:** a Faculty of Materials Science and Technology, Kim Chaek University of Technology PO Box 76 Pyongyang Democratic People's Republic of Korea jsh87927@star-co.net.kp

## Abstract

Cu-coated graphene-reinforced aluminum matrix composites (AMCs) are studied using first-principles calculations. Using Cu-coated graphene, the reinforced AMCs exhibit 1.5 times stronger interfacial bonding than the non-Cu-coated derivative due to hybridization between the 3d orbital of Cu and π orbital of graphene. Our work provides atomic insight into improved interfacial-bonding strength and electronic structure properties of graphene-reinforced AMCs by Cu-coating.

## Abstract 2

Cu-coated graphene-reinforced aluminum matrix composites (AMCs) exhibit 1.5 times stronger interfacial bonding than the non-Cu-coated derivative due to hybridization between the 3d orbital of Cu and π orbital of graphene.

## Graphical abstract

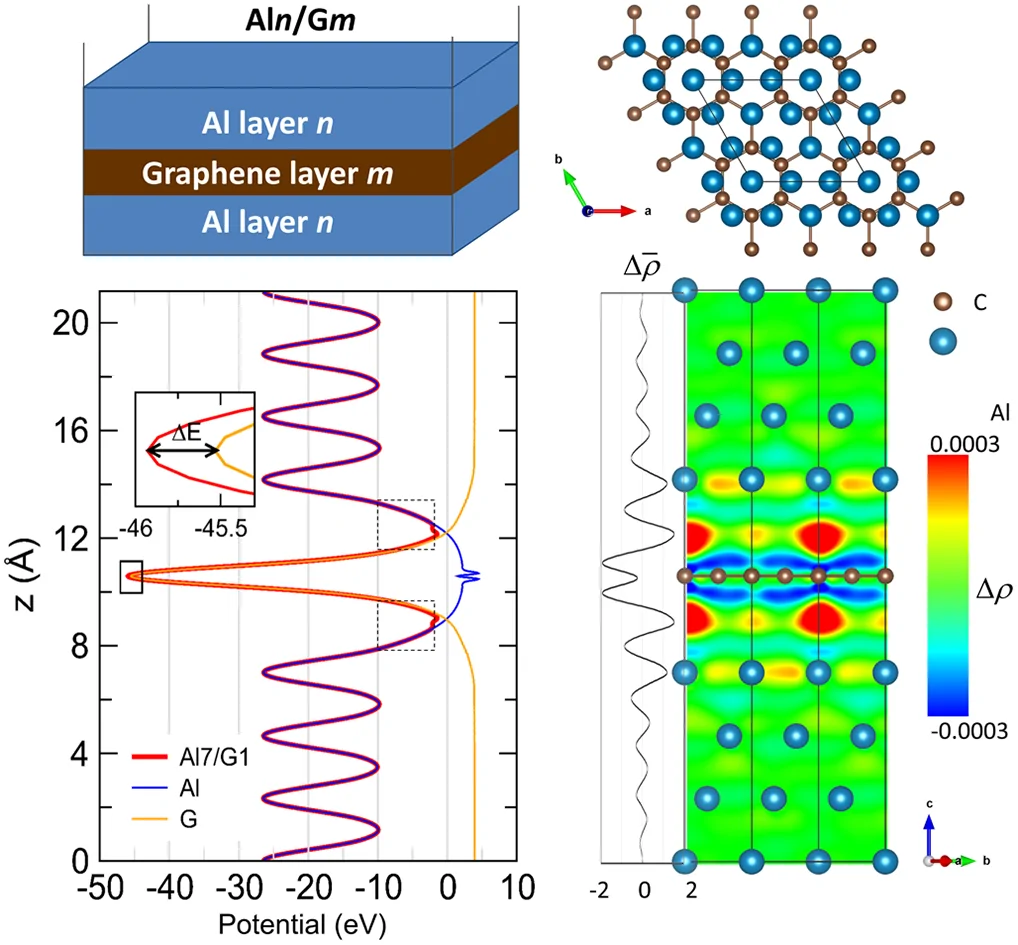

Graphene, a typical two-dimensional material with a honeycomb structure, has been widely studied since its isolation in 2004, being widely used as a reinforcing phase in metal matrix composites (MMCs) due to its high fracture strength and Young's modulus, and superior thermal conductivity.^1–6^ Among the MMCs, graphene-reinforced aluminum matrix composites (AMCs) have attracted great attention for use in power transmission devices, and automotive and aerospace industries due to their superior mechanical strength, thermal and electric conductivity, light weight and low cost compared to other aluminum alloys.^7–11^ However, the low wettability and dispersibility^12,13^ are great challenges for large-scale application of graphene-reinforced AMCs^8^ as they cause poor Al/graphene (Al/G) interfacial adhesion, resulting in deterioration of mechanical, thermal and transport properties.^14,15^ In order to strengthen the Al/G interface adhesion, various strategies of forming structural defects, adding functional groups and coating other metals on graphene^15–18^ have been employed, significantly enhancing the bond between metal and graphene. Among them, metal coating is regarded as the most effective method for improving wettability and dispersibility without changing the intrinsic structure of graphene. Because of the similar lattice parameters of Cu (Ni) and graphene, Cu and Ni have been pre-coated on graphene and subsequently improved the interfacial-bonding strength. In particular, Cu coating on graphene reduces the density mismatch between graphene and Al, and thus effectively prevents the graphene from floating upward during the stirring process.^19^ Moreover, Cu coating on graphene prevents aggregation of graphene and evolution of the Al_4_C_3_ phase,^14,20^ increasing the interfacial bonding intensity between the reinforcement and Al matrix. In this regard, *ab initio* calculations have been performed in order to provide theoretical insights into the improved interfacial-bonding strength and electronic transport properties of graphene-reinforced AMCs by Cu coating.^21–27^ However, most investigations employed a slab model where metal atoms were substituted or adsorbed on graphene, which limits quantitative understanding of the bulk properties for Cu-coated graphene-reinforced AMCs.

In this work, we investigated Cu-coating effects on interfacial-bonding strength, and mechanical and electronic properties by varying the number of intercalated graphene layers in Cu-coated graphene-reinforced AMCs (Al/Cu/G_*n*_, where *n* is the number of graphene layers). The Cu-coated graphene (Cu/G_*n*_) was constructed by stacking a layer of Cu(1 1 1) surface on both sides of the (2 × 2) graphene supercell with a lattice mismatch of less than 3.5%. The Al/Cu/G_*n*_ superlattice model was composed of a relaxed Cu/G_*n*_ surface and 
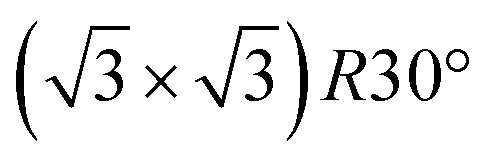
 supercells with seven layers of face-centered Al(1 1 1) surface^28^ (Fig. S1, SI), providing a lattice mismatch of −0.44–0.16%. With this model, the interfacial-bonding strength and formation energy, elastic constant tensor, phonon dispersion, energy band structure and density of states (DOS) were investigated based on state-of-the-art density functional theory (DFT) calculations. All the calculations were performed using the pseudo-potential plane-wave method as implemented in QUANTUM ESPRESSO (QE) 7.3.^29^ Elastic constants were calculated within the strain–stress method using ELASTIC code^30^ in combination with the QE package. Phonon dispersions and DOS were calculated considering anharmonic phonon–phonon interactions at finite temperature within the self-consistent phonon (SCP) theory, as implemented in ALAMODE code.^31^

First, we performed structural optimization for fcc-Cu with the *Fm*3̄*m* space group, and Cu/G_*n*_ and Al/Cu/G_*n*_ superlattices (Table S1, SI). Previous calculation results for fcc-Al and graphene superlattices were used for modeling the Al/G_*n*_ superlattice.^28^ For comparison of wettability of the Cu-coated and uncoated composites, we calculated formation energies *E*_f_ for Al bulk and Cu/G_*n*_ surfaces, and interface binding energies *E*_b_ for Al/Cu and Cu/G interfaces. We found that *E*_f_ varies slightly from −4.705 eV for Al/Cu/G_1_ to −4.732 eV for Al/Cu/G_5_ when increasing graphene content *p*_g_ (inset in Fig. 1(a)). The negative value of *E*_f_ indicates that the formation of Al/Cu/G_*n*_ superlattices from the Al bulk and Cu/G_*n*_ surface is an exothermic process. Moreover, the increment in magnitude of *E*_f_ implies improved formability with increasing *p*_g_. Then, the interlayer binding energy of the Al/Cu interface in the Al/Cu/G_*n*_ superlattices was calculated, showing that strong bonding between the Al layer and Cu layer was formed (see Table S2, SI). Furthermore, the interlayer binding energy of the Cu/G interface increased from −0.89 J m^−2^ for Al/Cu/G1 to −1.406 J m^−2^ for Al/Cu/G5 (Fig. 1(a)), which is ∼1.5 times larger than the binding energy between the Al layer and graphene layer in Al/G_*n*_ without Cu coating. Meanwhile, the negative values of *E*_b_ for all the Al/Cu/G_*n*_ composites demonstrate that the bond between graphene and Cu is still strong in the Al/Cu/G_*n*_ superlattice formation from the Cu/G_*n*_ surface and Al bulk. To intuitively show the electron transfer and charge redistribution upon the formation of the Cu/G interface, we calculated the electron density difference and its planar average. As shown in Fig. 1(b), the charge accumulation is remarkable in the middle part of the Cu/G interface and charge depletion is identified near graphene and Cu. The planar averaged charge density difference (Fig. 1(b)) is approximately 3 times larger than that in Al/G_*n*_,^28^ indicating that interfacial bonding between graphene and the metal layer becomes stronger than that in Al/G_*n*_, resulting in a larger potential barrier for electron transfer. Consequently, we find that the Cu-coating on graphene can significantly improve the wettability in graphene-reinforced AMCs, and also prevent graphene from floating upward during the stirring process due to the increased density of graphene.

**Fig. 1:**
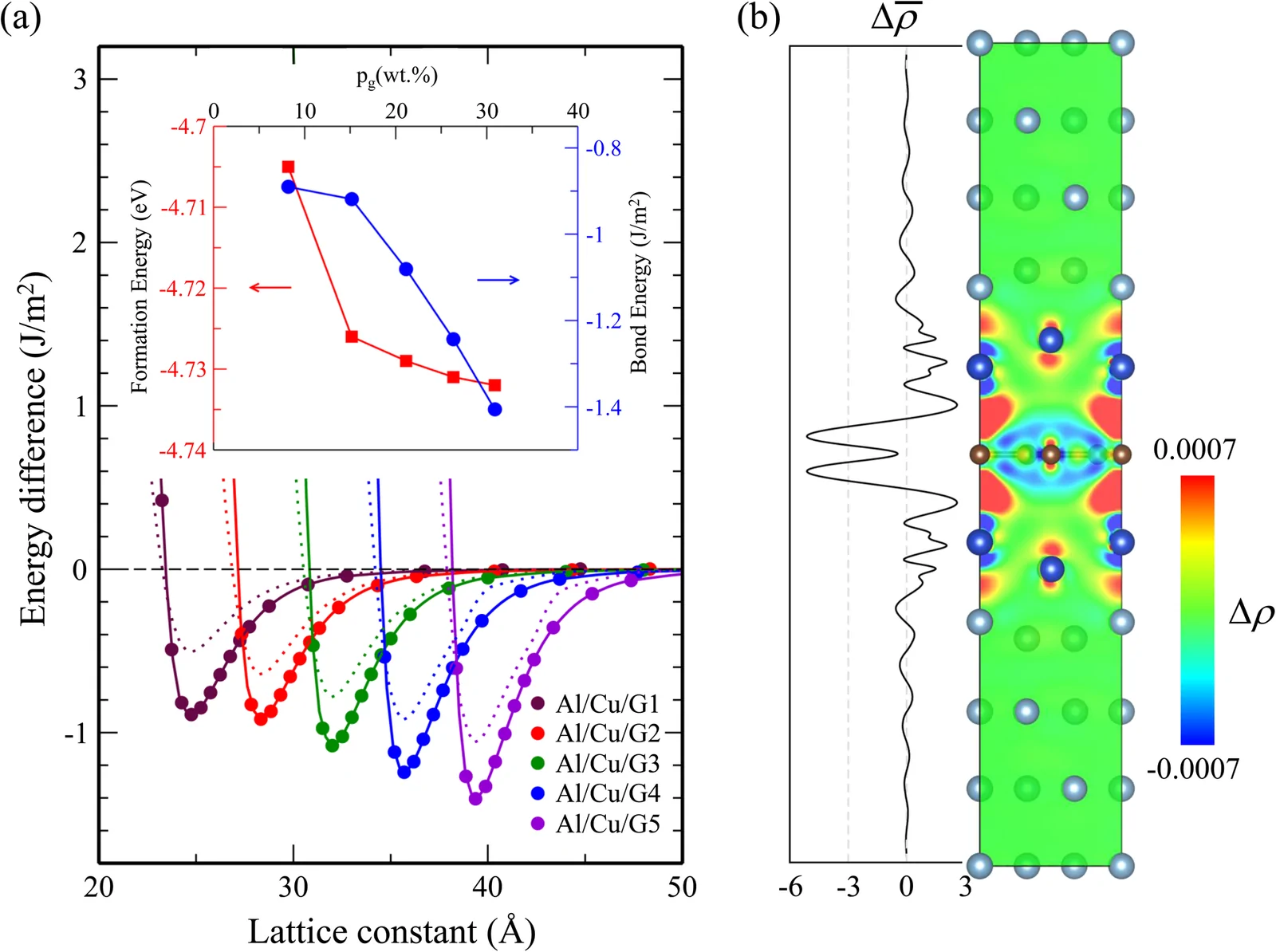
(a) Energy difference *versus* interlayer distance for Al/Cu/G_*n*_ (solid) and Al/G_*n*_ (dashed). The inset shows interface binding energy and formation energy as functions of graphene content (*p*_g_) in Al/Cu/G_1_. (b) Side view of Al/Cu/G_1_ with the isosurface plot of electron density difference (right panel) and planar average (left panel). Grey, blue and brown balls represent Al, Cu and C atoms.

Next, we studied the mechanical properties of the Al/Cu/G_*n*_ AMCs by calculating the elastic stiffness constant *C*_*ij*_. The six elastic constants (*C*_11_, *C*_12_, *C*_13_, *C*_33_, *C*_44_ and *C*_66_) satisfy the well-known Born stability criteria (see Table S3, SI), proving the mechanical stability of Al/Cu/G_*n*_. Fig. 2 shows the *C*_*ij*_, elastic moduli, and Poisson's and Pugh's ratios calculated when varying the graphene content, with the available experimental data being used for fcc-Al^32^ and graphene.^33^ In Fig. 2, the point where the graphene content is 0% corresponds to the elastic constant of pure aluminum, and the point where it is 100% corresponds to the elastic constant of pure graphene. At a glance, the planar elastic constants *C*_11_ and *C*_12_ show a tendency to increase with graphene content; in particular, the principal elastic constant *C*_11_ increases rapidly with increasing graphene content, from 274.4 GPa for Al/Cu/G_1_ to 543.1 GPa for Al/Cu/G_5_, as shown in Fig. 2(a). However, it is considerably lower than the mechanical strength of graphene. On the other hand, components perpendicular to the interface (*C*_33_, *C*_13_ and *C*_44_) decrease with increasing *p*_g_, illustrating that the resistance to deformation along the *a*-axis becomes stronger, but that along the *c*-axis becomes weaker as the number of graphene layers increases (see Table S3, SI). The elastic constant *C*_33_ slightly increases compared to that of Al/G_*n*_, which is attributed to the stronger bonding between Cu and graphene. This result shows that Cu-coated graphene is a better reinforcement for AMCs than pure graphene. We also calculated the bulk (*B*), shear (*G*) and Young's (*E*) moduli for the polycrystalline solids. These quantities also increase with the increment in graphene content (Fig. 2(b)). In addition, we calculated Pugh's ratio (*µ*) and Poisson's ratio (*ν*) to determine the critical value of graphene content at which the composite transitions from ductile to brittle. From our calculations, we predicted that the critical value of the graphene content is 14.98 (15.15)% for Pugh's (Poisson's) ratio. It can be seen that the critical value of the graphene content for maintaining ductility of the Al/Cu/G composite is 14.98%, which is lower than that of the non-Cu-coated composite (21.75%).

**Fig. 2:**
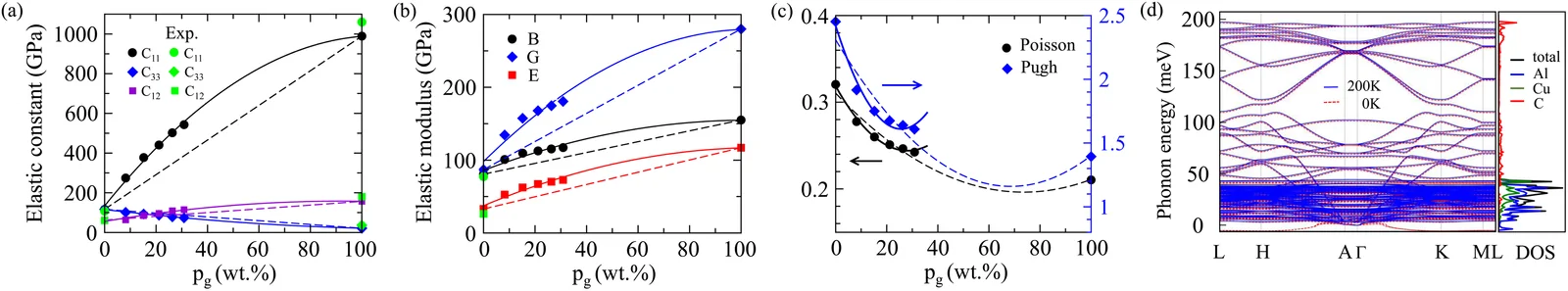
(a) Elastic stiffness constants, (b) bulk (*B*), shear (*G*) and Young's (*E*) moduli, and (c) Poisson's and Pugh's ratios for Al/Cu/G_*n*_. Solid (dashed) lines show quadratic (linear) fitting results. (d) Phonon dispersion curves and atom-resolved phonon DOS in Al/Cu/G_1_ at 0 and 200 K.

To evaluate the thermodynamic stability of Al/Cu/G_1_, lattice dynamics calculations have been carried out at finite temperatures by considering the anharmonic phonon–phonon interactions. As shown in Fig. 2(d), the phonon dispersion curves at 0 and 200 K did not differ significantly in the high-energy optical modes, while soft phonon modes with negative energy were identified at 0 K. As can be seen from the atom-resolved phonon DOS, the negative phonon modes are mainly ascribed to the atomic vibrations of Al. However, the negative phonon eigenvalues were renormalized to become positive values at 200 K, pointing to the Al/Cu/G_*n*_ superlattice being dynamically stable at elevated temperatures. It was found that the Al and Cu atoms have dominant contributions to the acoustic and low-energy optical modes, while the high-energy optical modes are dominated by the C atoms of graphene. This indicates that the Al and Cu atoms play an important role in lattice thermal transport properties in the Al/Cu/G_*n*_ composite.

In order to understand the nature of the interfacial-bonding between graphene and the Cu-coating layer, and provide atomic insight into the electronic structure and transport properties, we calculated the atom-projected energy band structures and local density of states (LDOS) for the Al/Cu/G_*n*_ composite. As shown in Fig. 3(a), unlike in the Al/G_*n*_ superlattice, the intrinsic π-band shape of graphene disappears in the energy range from −2 eV to −3 eV below the Fermi level, which is due to the hybridization between the 3d state of Cu and the 2p_*z*_ state of the C atoms. This is clearly evidenced in the partial DOS in Fig. 3(a) and the integrated LDOS (iLDOS) in Fig. 3(b). Orbital hybridization results in a weak state that electrons can occupy between the Cu layer and the graphene layer in the vicinity of −3 eV below the Fermi level in the LDOS (Fig. 3(c)). This is in contrast with the result that the density of states between the Al layer and graphene layer is almost zero in this energy range for Al/G composites.^28^ Thus, the weak physical bonding in Al/G_*n*_ is formed by charge redistribution, whereas in Al/Cu/G, interfacial bonding is formed by electron orbital hybridization, providing a stronger interfacial bonding than the weak physical bonding. On the other hand, in the energy band structure, no π band gap opening was found, as in some graphene composites. The π band gap opening does not occur for a metal/G interface with physical bonding, but for a metal/G interface with chemical bonding. Nevertheless, the absence of band opening in the Al/Cu/Gn composite is due to the formation of orbital hybridization only in the energy range around the 3d band of Cu. As shown in Fig. 3(a), the density of states of the Cu 3d electron increases rapidly with decreasing energy from 1 eV below the charge neutral level of graphene. Therefore, the intrinsic π band shape of graphene does not change near the Dirac cone point, only downward from −1 eV below it. This indicates that the high electrical conductivity and electron thermal conductivity of graphene in Al/Cu/G composites can be retained, *i.e.*, the graphene can make a significant contribution to the electrical and thermal conductivity in the direction parallel to the interface. Also, in the energy band structure, the charge neutral level of graphene and the Fermi level of the composite are not coincident, because the Dirac cone point of graphene is shifted down. This indicates that graphene is n-doped in Al/Cu/G composites. In fact, according to the conventional Schottky–Mott model, graphene being n-type doped in contact with Cu (∼5.2 eV for the work function) and graphene (∼4.6 eV for the work function) cannot be explained. Such doping behavior can be explained phenomenologically by Pauli's repulsive force.^34^ Since the interlayer distance at the Cu/G interface is less than that at the Al/G interface, orbital hybridization between the 3d electrons of Cu and the π electrons of graphene is formed. Then, the Cu 4s orbital with a large radius overlaps with the π orbital of graphene, which is energetically unstable, and a strong Pauli repulsion occurs. As a result, the Dirac cone of graphene is shifted down. Because of the small interlayer distance of the Cu/G interface, the repulsive force between the 4s electron of Cu and the π electron of graphene is larger than that between the 3s electron of Al and the π electron of graphene, so that the Dirac cone point is shifted down more than that of Al/G_*n*_. This trend is also evident in the planar averaged electronic potentials of Al/Cu, graphene and Al/Cu/G_1_ in Fig. 3(d). To evaluate the relative shift in the energy level of graphene, a uniform reference vacuum energy was used in the planar averaged potential lineups. The potential of graphene was shifted down more in Al/Cu/G than in Al/G^28^ (see Table S5, SI). Also, the potential difference decreases from 465.4 meV to 369 meV with an increasing number of graphene layers, which is related to the fact that the interaction between the Cu layer and graphene layer weakens with the number of layers.

**Fig. 3:**
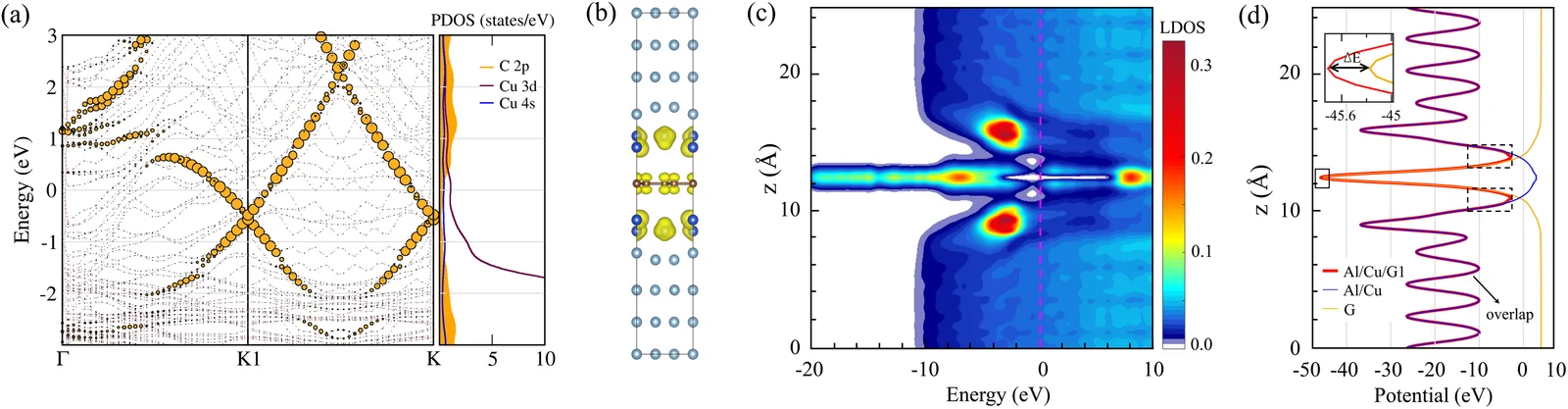
(a) Atom-projected band structure of Al/Cu/G_1_ for a graphene (2 × 2) supercell and partial DOS of Al/Cu/G_1_. The size of the orange-colored circles indicates the amount of contribution for graphene π-orbital components. (b) Isosurface plot for integrated local density of states with energies ranging from −3 eV to −2 eV. (c) Local density of states in Al/Cu/G_1_ along the *z*-axis. (d) Planar averaged electrostatic potential lineups of Al/Cu/G_1_, Al/Cu and graphene along the *z*-axis. The Fermi level is set to zero.

Finally, for considering the tunnelling transmission probability through the potential barrier at the interface, we assumed a one-dimensional parabolic potential barrier with a height of 9.98 eV and a width of 1.59 Å (dashed rectangle in Fig. 3(d)). The height of the barrier at the Cu/G interface is larger than that at the Al/G interface without Cu coating (see Table S5, SI). Such a high barrier makes the charge transfer from Cu to the graphene layer more difficult than in Al/G. However, the width of the barrier at the Cu/G interface is relatively small, because the interlayer distance at the Cu/G interface is less than at Al/G. This, in contrast to the above, makes the charge transfer relatively easy by allowing a further improvement in the tunnelling transmission of electrons at the Cu/G interface. Due to these two opposite effects, the tunnelling transmission probability^35^ (∼1.77%) does not differ significantly from that of the non-Cu-coated derivative (∼1.68%). This result shows that the tunnelling barriers can greatly reduce the carrier injection, because the Pauli repulsion force between the 4s electron of Cu and the π electron of graphene plays a dominant role in redistributing electron density. However, this finding is in good agreement with the experimental fact that the contact resistance between different metal and graphene contacts (including both physisorption and chemisorption of the metal) does not change significantly.^36^

In summary, by using Cu-coated graphene as a reinforcement, relatively weak chemical bonds were formed due to the hybridization between the 3d orbital of Cu and π orbital of graphene, but the bonding strength was stronger than the weak physical bonding in the Al/G interface. As a result, the interfacial bonding strength was about 1.5 times stronger than the non-Cu-coated derivative. Also, there was no significant difference in mechanical properties compared to the non-Cu-coated derivative, but the components perpendicular to the interface were slightly increased, and the critical value of graphene content in the composite to maintain ductility was reduced to 14.98%, from 21.75% in the Al/G composite. The phonon dispersion, energy band structure and LDOS of the Al/Cu/G composite were calculated in order to gain insight into the thermodynamic stability, thermal conductance and interfacial contact resistance of the composites.

## Author contributions

Sin-Hyok Jon developed the original project, performed the calculations and drafted the first manuscript. Jae-Bok Ri, Chol-Jong, Un-Gi Jong and Songchol Hong assisted with the DFT calculations and the post-processing of calculation results, and contributed to useful discussions. Sin-Hyok Jon and Un-Gi Jong supervised the work. All authors reviewed the manuscript.

## Conflicts of interest

The authors declare no competing of financial interest.

## Supplementary Material

RA-OLF-D6RA04391G-s001

## Data Availability

The data that support the findings of this study are available from the corresponding author upon reasonable request. Supplementary information (SI): crystalline structures for cubic Fe and hexagonal Mg and Be compound, charge density and ELF for Mg_2_FeH_6_ comparison of energy band structures of MgBeFeH_6_ and FeH_6_ framework, partial DOS and Helmholtz free energy for Mg2FeH6. See DOI: https://doi.org/10.1039/d6ra04391g.
