# Supplementary material for: Improved interfacial-bonding strength and electronic structure properties of graphene-reinforced Al composites by Cu coating
Source: RSC Adv. 2026 Oct 28;16(49):66819–23. doi: 10.1039/d6ra04391g (PMC13714721; doi:10.1039/d6ra04391g)
Supplement: Supplementary file 1 [file RA-016-D6RA04391G-s001.pdf]

Supplementary Information (SI) for RSC Advances.
This journal is © The Royal Society of Chemistry

Electronic Supplementary Information – Improved interfacial-bonding strength and electronic structure properties of graphene-reinforced Al composites by Cu-coating

Sin-Hyok Jon[a]*, Jae-Bok Ri[a], Chol-Jong[a], Un-Gi Jong[b], Songchol Hong[c]

[a]*Faculty of Materials Science and Technology, Kim Chaek University of technology, Central District, Pyongyang, DPR Korea.*
[b]*Faculty of Materials Science,* **Kim Il Sung** *University, Ryongnam-dong, Taesong District, Pyongyang, DPR Korea.*
[c]*Institute of nanoscience and technology, Kim Chaek University of technology, Central District, Pyongyang, DPR Korea.*

1. Structural optimization

All the calculations were performed using the pseudopotential plane-wave method as implemented in QUANTUM ESPRESSO(version 7.3) [1] within the density functional theory framework. For a description of the ion-valence electrons Columbic interaction, we used ultrasoft pseudopotentials from the GBRV library [2] (i.e. c.pbesol_v1.2.uspp.F.UPF, al_pbesol_v1.uspp.F.UPF and cu_pbesol_v1.2.uspp.F.UPF). Here, the valence electronic configurations of the atoms are as follow: Al:$3s^2 3p^1$; C:$2s^2 2p^2$ Cu:$3s^2 3p^6 4s^1 3d^{10}$. The Perdew-Burke-Ernzerhof(PBE) functional [3] within the generalized gradient approximation (GGA) was used to describe the exchange correlation interaction between the valence electrons. The van der Waals(vdW) dispersion interaction between graphene layers was taken into consideration by appling the semiempirical DFT-D3 method [4]. Then empirical parameters were provided in the QE package. As the major computational parameters, the plane wave cutoff energies are set to be 60 Ry for the wave function and 650 Ry for the electron density, and the Monkhorst-Pack special points are set to be $(6 \times 6 \times 6)$ for Cu/Gn and Al/Cu/Gn SLs, $(8 \times 8 \times 8)$ for Cu bulk. The self-consistent convergence threshold for total energy is 10^{-9} Ry, and the convergence threshold for atomic force in structural relaxations is 5×10^{-4} Ry/Bohr and the lattice pressure was less than 0.05 kBar. For more accurate calculations of the electronic density of state (DOS) and electron density difference, we used a denser k-points mesh of $(10 \times 10 \times 3)$. We apply the smearing approach method by Fermi-Dirac function with a 0.02 Ry Gaussian spreading for structural optimization. The visualization of crystalline lattice was performed using the VESTA code.

Table S1. The optimized lattice constants and angles of fcc-Cu bulk unit cell, Cu(1 1 1) (2×2) and Al/Cu/Gn SLs. The optimized lattice constants and experimental values of fcc-Al bulk unit cell, Al(1 1 1) surface and graphene are provided from [a]Ref. [5], [b]Ref. [6], [c]Ref. [7] and [d]Ref. [8].

	Lattice constant (Å)			Lattice angle (deg)		
	a	b	c	α	β	γ
fcc-Al[a]	4.0191	4.0191	4.0191	90	90	90
(Exp.[b])	(4.0496)	(4.0496)	(4.0496)	(90)	(90)	(90)
Al(1 1 1) $(\sqrt{3} \times \sqrt{3})R30°$[a]	4.9224	4.9224		90	90	60
fcc-Cu	3.6141	3.6141	3.6141	90	90	90
(Exp.[c])	(3.615)	(3.615)	(3.615)	(90)	(90)	(90)
Cu(1 1 1) (2×2)	5.1111	5.1111	5.1111	90	90	60
graphene (2×2)[a]	4.9320	4.9320	3.6831			60
(Exp.[d])	(4.92)	(4.92)	(3.40)			(60)
Cu/G1	4.9007	4.9007		90	90	60
Cu/G2	4.9130	4.9130		90	90	60
Cu/G3	4.9217	4.9217		90	90	60
Cu/G4	4.9282	4.9282		90	90	60
Cu/G5	4.9301	4.9301		90	90	60
Al/Cu/G1	4.9172	4.9172	24.7455	90	90	60
Al/Cu/G2	4.9219	4.9219	28.3366	90	90	60
Al/Cu/G3	4.9243	4.9243	32.0079	90	90	60
Al/Cu/G4	4.9258	4.9258	35.6936	90	90	60
Al/Cu/G5	4.9267	4.9267	39.3739	90	90	60

First, as a preliminary step, we performed the geometric optimization of Cu($Fm\bar{3}m$) and Cu/Gn. The geometric optimiza-

*Email: jsh87927@star-co.net.kp

tion of aluminum ($Fm\bar{3}m$) and graphene ($P6/mmm$) was carried out in our previous work [5]. The Cu/Gn superlattice was fabricated on the Cu coated graphene surface by stacking a layer of Cu(1 1 1) (2×2) surface to both sides of the graphene (2×2) cell and geometry optimization was carried out. Then, the lattice parameter c perpendicular to the plane of graphene was set to 20 Å to eliminate the interaction between the planes. Then, a layer of Cu(1 1 1) (2×2) surface was joined on both sides of the graphene (2×2) cell to create a Cu coated graphene surface structure and perform the geometry optimization. The Al/Cu/Gn superlattice was composed of graphene (2×2) cells and Al(1 1 1) $(\sqrt{3} \times \sqrt{3})R30^\circ$ surface cells [5] and Cu coated graphene surface cells to minimize lattice mismatch. Then, the lattice mismatch ratio is $-0.44 \sim 0.16\%$ (see Fig. S1). In the Al/Cu/Gn superlattice cell, the number of Al layers set to 7, to 2 on Cu layer and the number of graphene layers intercalated between Cu layers increased from 1 to 5. The weight ratio of graphene in Al/Cu/Gn composites varies from 8.2% to 30.9%. After structural optimization, the superlattice has hexagonal crystalline structure (space group number 149-$P312$). Table S1 shows the optimized lattice parameters of fcc-Al, fcc-Cu bulk cells, graphene cells, Cu/Gn surface cells and supercells of Al/Cu/Gn superlattices.

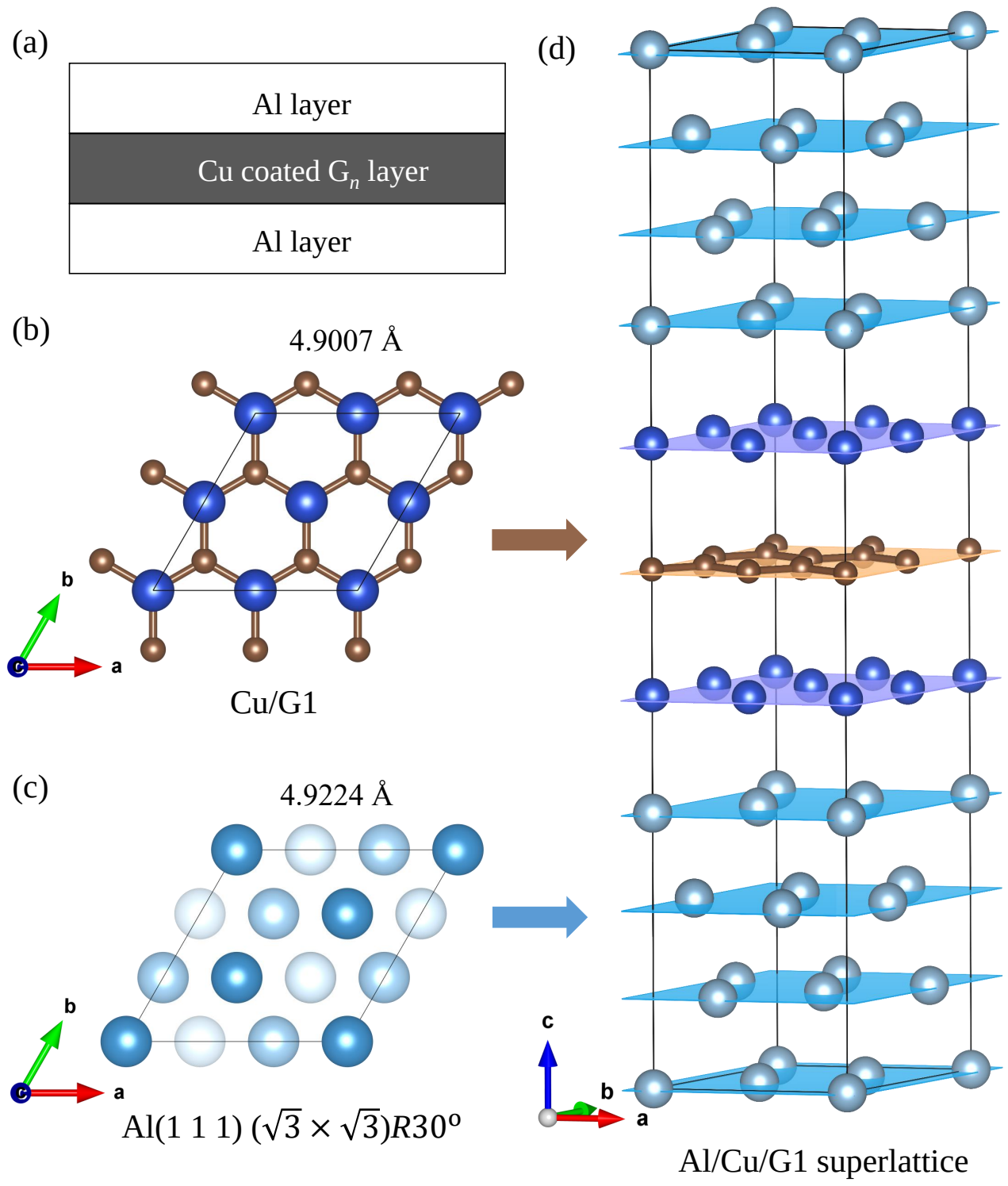

Fig. S1 (a) Sketch of Al/Cu/Gn superlattice structure. Top view of (b) Cu/G1 surface unit cell and (c) $(\sqrt{3} \times \sqrt{3})R30^\circ$ cell of Al(1 1 1) surface. (d) Perspective view of Al/Cu/G1 superlattice composed of 7 Al layers of Al(1 1 1) $(\sqrt{3} \times \sqrt{3})R30^\circ$ surface cell and Cu/G1 surface. Blue-colored big balls represent the Cu atoms and brown-colored small balls indicate the C atoms

2. Interface formation energy and bonding strength

We calculated the formation energy and the binding energy to evaluate the formability and wettability. The formation energy was estimated by the following equation,

$$E_f = E^{\text{SL}}_{\text{Al/Cu/G}n} - E^{\text{surf}}_{\text{Cu/G}n} - \frac{n_{\text{Al}}}{4} E^{\text{fcc}}_{\text{Al}}, \tag{S1}$$

where $E^{\text{SL}}_{\text{Al/Cu/G}n}$ is the total energy of the optimized superlattice unit cell, $E^{\text{surf}}_{\text{Cu/G}n}$ is the energy of the relaxed Cu/Gn surface unit cell, and $E^{\text{fcc}}_{\text{Al}}$ is the energy of the optimized fcc Al unit cell. n_{Al} is the number of Al atoms in the superlattice, i.e., it is equal to 21. The interfacial binding energy of Al/Cu/Gn superlattices was evaluated by the following equation,

$$E_{b(\text{Al/Cu})} = \frac{1}{n_{\text{inter}} S_{\text{inter}}} \left[E^{\text{SL}}_{\text{Al/Cu/G}n}(d_{\text{Al/Cu}} = d_{\text{eq}}) - E^{\text{SL}}_{\text{Al/Cu/G}n}(d_{\text{Al/Cu}} = d_{\infty}) \right], \tag{S2}$$

$$E_{b(\text{Cu/G}n)} = \frac{1}{n_{\text{inter}} S_{\text{inter}}} \left[E^{\text{SL}}_{\text{Al/Cu/G}n}(d_{\text{Cu/G}n} = d_{\text{eq}}) - E^{\text{SL}}_{\text{Al/Cu/G}n}(d_{\text{Cu/G}n} = d_{\infty}) \right], \tag{S3}$$

Here, $E_{b(\text{Al/Cu})}$ and $E_{b(\text{Cu/G}n)}$ are interfacial binding energy per atom of Al/Cu interface and Cu/Gn interface, respectively, in Al/Cu/Gn superlattices. n_{inter} and S_{inter} are the number and the area of equivalent interface, respectively, i.e., n_{inter} is equal to 2. $E^{\text{SL}}_{\text{Al/Cu/G}n}$ $(d_{\text{Al/Cu}} = d_{eq})$ and $E^{\text{SL}}_{\text{Al/Cu/G}n}$ $(d_{\text{Cu/G}n} = d_{eq})$ are the total energy of the Al/Cu/Gn superlattice with the

Table S2. Calculated formation energy and interfacial binding energy of Al/Cu/Gn SLs.

Compound	$E^{\rm SL}_{{\rm Al/Cu/G}n}$ (Ry)	$E^{\rm surf}_{{\rm Cu/G}n}$ (Ry)	$E^{\rm fcc}_{\rm Al}$ (Ry)	E_f (eV)	$S_{\rm inter}$(Å^2)	$E_{b({\rm Cu/G}n)}$ (J/m^2)	$E_{b({\rm Al/Cu})}$ (meV)
Al/Cu/G1	−3463.47326	−3321.97106	−26.88676	−4.705	10.470	−0.89034	−224.61
Al/Cu/G2	−3555.44141	−3413.93767		−4.726	10.490	−0.91871	−186.47
Al/Cu/G3	−3647.40991	−3505.90592		−4.729	10.500	−1.08099	−159.19
Al/Cu/G4	−3739.37850	−3597.87437		−4.731	10.506	−1.24340	−135.28
Al/Cu/G5	−3831.34712	−3689.84294		−4.732	10.510	−1.40595	−127.62

equilibrium interlayer distance in Al/Cu interface and Cu/Gn interface, respectively, i.e., the total energy of the optimized Al/Cu/Gn superlattice. $E^{\rm SL}_{{\rm Al/Cu/G}n}$ ($d_{\rm Al/Cu} = d_\infty$) and $E^{\rm SL}_{{\rm Al/Cu/G}n}$ ($d_{{\rm Cu/G}n} = d_\infty$) are the total energy of the Al/Cu/Gn superlattices with the interlayer distance at d_∞(15 Å) in Al/Cu interface and Cu/Gn interface, respectively. The interfacial binding energy of Cu/Gn interface is calculated to compare the interfacial bonding strength between metal layer and graphene layer in Al/Gn and Al/Cu/Gn composites. To evaluate the change of binding energy with varying the interlayer distance between Cu layer and graphene layer, the atomic positions in the optimized superlattice structure were fixed, and the superlattice energy was calculated by varying only the interlayer distance. Next, to evaluate the charge transport and redistribution upon interface formation, we calculated the electron density difference by $\Delta\rho(\boldsymbol{r}) = \rho^{\rm SL}_{{\rm Al/Cu/G}n}(\boldsymbol{r}) - \rho^{slab}_{\rm Al/Cu}(\boldsymbol{r}) - \rho^{slab}_{{\rm G}n}(\boldsymbol{r})$. where $\rho^{\rm SL}_{{\rm Al/Cu/G}n}(\boldsymbol{r})$ is the spatial electron density of the Al/Cu/Gn superlattice, $\rho^{slab}_{\rm Al/Cu}(\boldsymbol{r})$ and $\rho^{slab}_{{\rm G}n}(\boldsymbol{r})$ are the spatial electron densities of the Al/Cu and Gn slabs with the fixed atomic positions, respectively. In addition, the planar average of electron density difference parallel to the interface is also calculated with $\Delta\bar{\rho}(z) = \iint_A \Delta\rho(\boldsymbol{r})dxdy$, where A is the area of the interface.

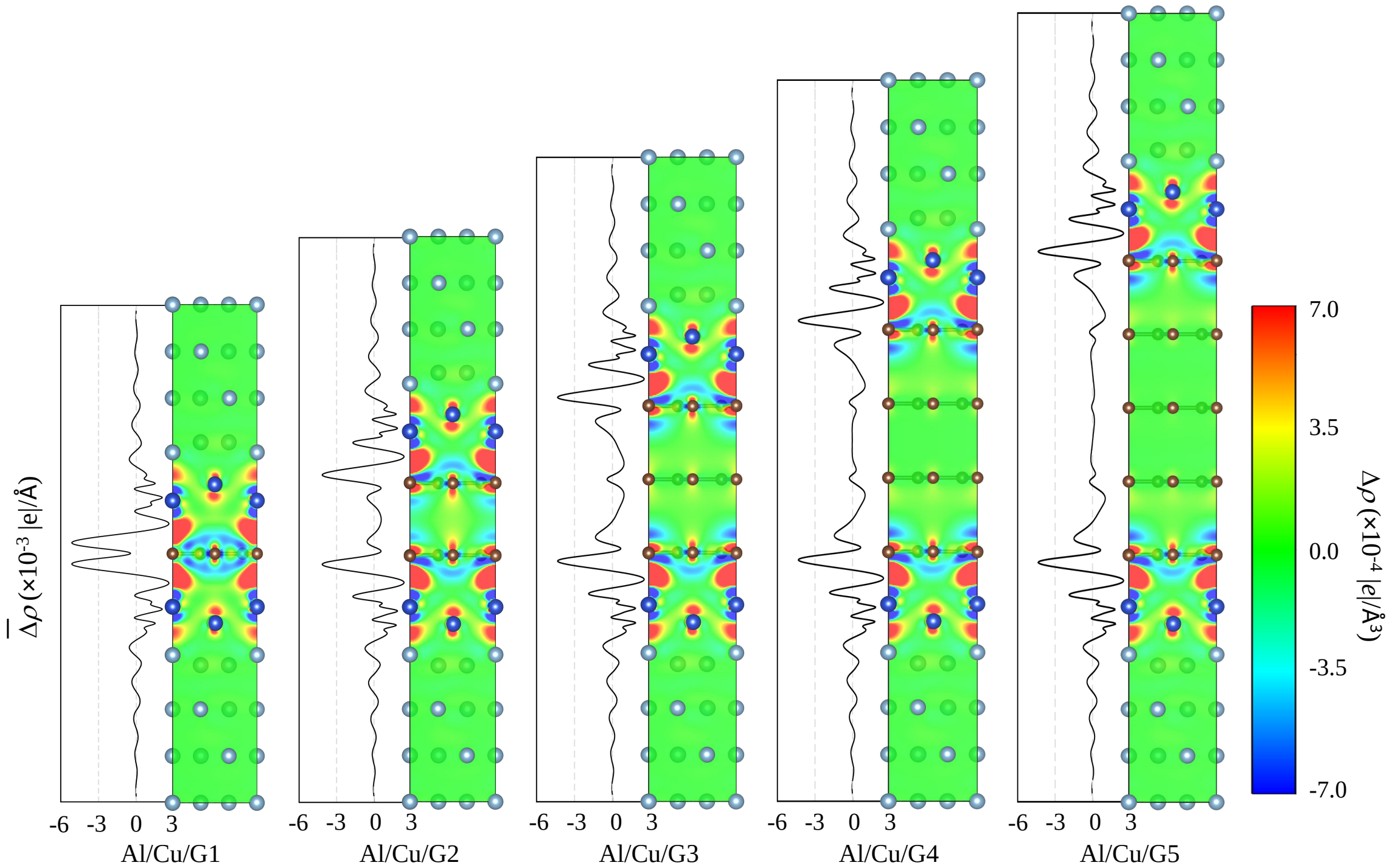

Fig. S2 Planar averaged electron density difference as a function of z position (left panel) and its isodensity plot projected on (1 0 0) plane in Al/Cu/Gn superlattices. Positive (negative) value indicates electron gain (loss).

3. Elastic constants and mechanical properties

The elastic constants were calculated the by strain-stress method using the ELASTIC code[7] in connection with the QE package. The maximum Lagrangian strain was set to be 0.002 and the number of the distorted structures was set to be 13. Because the Al/Cu/Gn is hexagonal crystal, which has six different elastic constants, C_{11}, C_{12}, C_{13}, C_{33}, C_{44}, C_{66}, etc., but here only five elastic constants are independent, since $C_{66} = (C_{11} - C_{12})/2$. The mechanical stability of the superlattices can be checked by the Born criteria as follows.

$$C_{11} > 0,\ C_{11} - C_{12} > 0,\ C_{44} > 0,\ C_{66} > 0,\ (C_{11} + C_{12})C_{33} - 2C_{13}^2 > 0 \tag{S4}$$

Using the elastic constants, the bulk modulus(B) and shear modulus(G) of the hexagonal system are calculated by the following equation in the Voigt(V) and Reuss(R) approximations.

$$B_{\rm V} = \frac{2(C_{11} + C_{12}) + C_{33} + 4C_{13}}{9},\quad G_{\rm V} = \frac{C_{11} + C_{12} + 2C_{33} - 4C_{13} + 12C_{44} + 12C_{66}}{30} \tag{S5}$$

Table S3. Calculated elastic stiffness constants for the fcc-Al bulk, Al/Cu/Gn and Al/Gn superlattices, and graphene. The experimental values are provided for fcc-Al from [a]Ref. [9] and for graphite from [b]Ref. [10]. The calculated value for fcc-Al bulk, Al/Gn superlattices and graphene are provided from our previous research (Ref. [5])

Compound	Elastic constants (GPa)					
	C_{11}	C_{12}	C_{13}	C_{33}	C_{44}	C_{66}
fcc-Al	116.8	62.6			36.8	
(Exp.[a])	(106.75)	(60.41)			(28.34)	
Al/Cu/G1	274.4	66.5	57.5	101.7	26.9	105.5
Al/G1	283.9	83.5	33.6	97.6	21.9	100.2
Al/Cu/G2	377.5	85.1	48.3	93.1	23.7	146.2
Al/G2	393.8	98.3	25.4	88.7	18.0	147.8
Al/Cu/G3	440.9	96.2	39.6	86.7	20.7	177.3
Al/G3	474.9	110.1	19.8	82.1	15.6	182.4
Al/Cu/G4	502.5	109.6	31.4	78.7	17.1	193.4
Al/G4	528.0	109.4	22.6	63.8	11.9	209.3
Al/Cu/G5	543.1	114.8	27.8	74.0	15.3	214.2
Al/G5	579.3	118.1	17.4	61.9	10.5	230.6
Graphene	989.1	156.8	−1.1	21.3	−2.4	416.2
(Exp.[b])	(1060)	(180)	(15)	(36.5)	(0.3)	(440)

$$B_{\mathrm{R}} = \frac{(C_{11}+C_{12})C_{33}-2C_{13}^2}{C_{11}+C_{12}+2C_{33}-4C_{13}}, \quad G_{\mathrm{R}} = \frac{5}{2}\frac{[(C_{11}+C_{12})C_{33}-2C_{13}^2]C_{44}C_{66}}{3B_{\mathrm{V}}C_{44}C_{66}+[(C_{11}+C_{12})C_{33}-2C_{13}^2](C_{44}+C_{66})} \tag{S6}$$

The Hill(H) approximations are obtained as arithmetic mean values from the Voight and Reuss approximations.

$$B_{\mathrm{H}} = \frac{1}{2}(B_{\mathrm{V}}+B_{\mathrm{R}}), \quad G_{\mathrm{H}} = \frac{1}{2}(G_{\mathrm{V}}+G_{\mathrm{R}}) \tag{S7}$$

Then, the Young's modulus(E), Poisson's ratio(ν) and Pugh's ratio(μ) were calculated as follows,

$$E = \frac{9BG}{3B+G}, \quad \nu = \frac{3B-2G}{6B+2G}, \quad \mu = \frac{B}{G} \tag{S8}$$

Table S4 shows the calculated elastic moduli, Poisson's and Pugh's ratio for the composites.

Table S4. Calculated elastic moduli of Al/Cu/Gn superlattices within the Voigt (V), Reuss (R) and Hill (H) approximations. The calculated values are provided for fcc-Al and graphene from our previous research ([a]Ref. [5]). The experimental values are provided for fcc-Al from [b]Ref. [9].

Compound	Bulk modulus (GPa)			Shear modulus (GPa)			Young's modulus (GPa)			Poisson	Pugh
	V	R	H	V	R	H	V	R	H	ν	μ
fcc-Al[a]	80.68	80.68	80.68	32.91	32.18	32.54	86.90	85.20	86.05	0.3222	2.4794
(Exp[b])	(77.3)			(26.2)	(25.9)				(70.2)	(0.35)	
Al/Cu/G1	112.61	89.27	100.94	62.82	42.54	52.68	158.90	110.13	134.52	0.2777	1.916
Al/Cu/G2	134.61	84.29	109.45	83.15	41.93	62.54	206.85	107.89	157.37	0.2600	1.750
Al/Cu/G3	146.59	78.66	112.63	95.62	38.83	67.22	235.63	100.02	167.83	0.2511	1.675
Al/Cu/G4	158.72	71.75	115.24	106.88	33.71	70.30	261.87	87.43	174.65	0.2465	1.639
Al/Cu/G5	166.78	67.86	117.32	114.94	30.95	72.94	280.40	80.59	180.49	0.2425	1.608
Graphene[a]			138.51			99.43			240.70	0.2104	1.393

4. Electronic band structure and density of states

The projected band structure of the superlattice was calculated along Γ−K1−K path with high symmetry in first Brillouin zone of graphene (2×2). Here K1 point is equivalent to K point of (1×1) unit cell in 1BZ. We have analyzed the contribution of the π orbital to graphene in the band structure projected onto the atomic orbitals. Next, the projected density of state (PDOS) was calculated. Figure S3 shows DOS of C 2p state, Cu 3d state and Cu 4s state in the unit of states per eV. The DOS of other states was not shown because of the very small value in the energy region near the Fermi level.

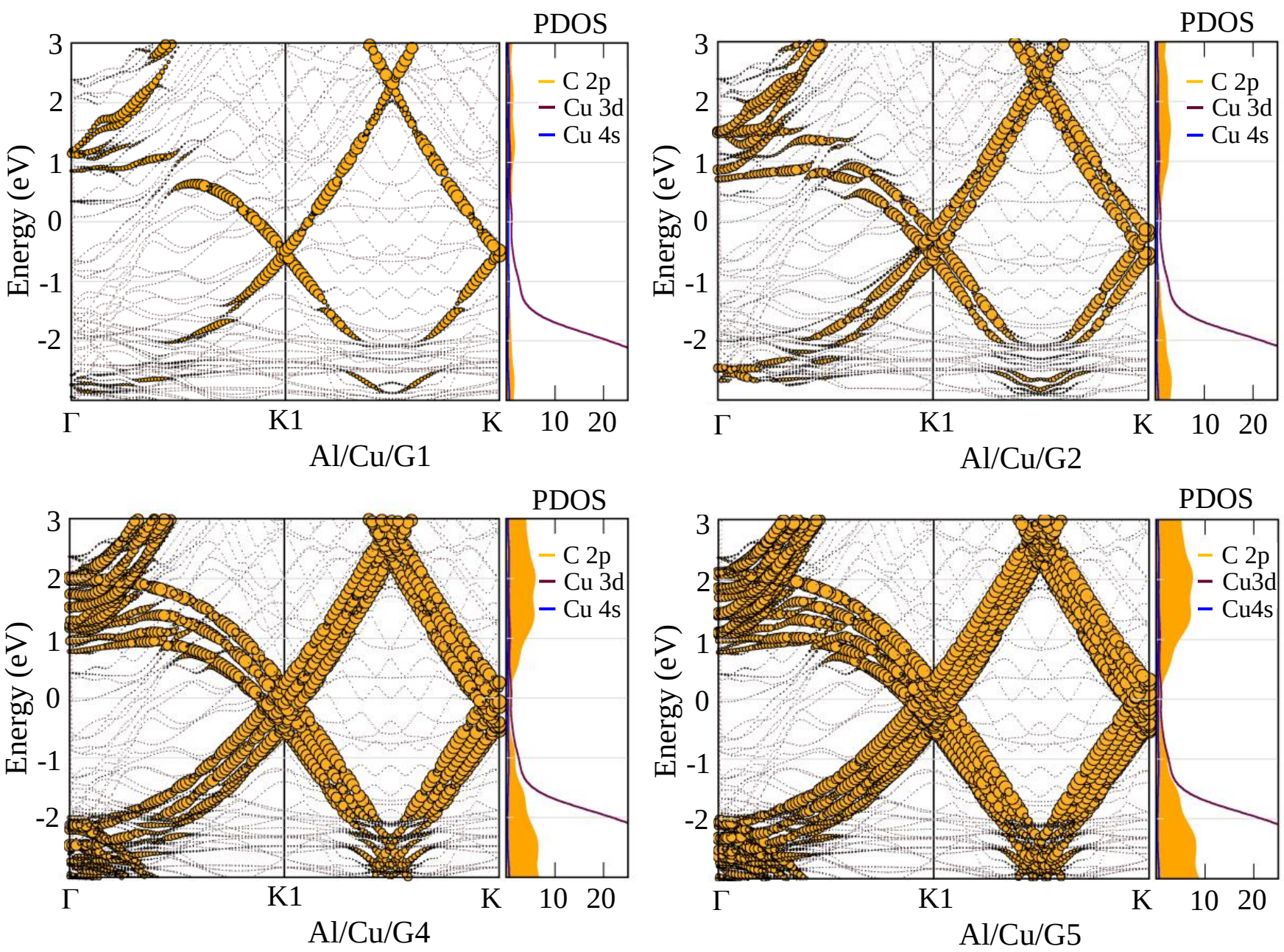

Fig. S3 Atom-projected band structures and PDOS of superlattices. The band structure was calculated along the path Γ−K1−K of the first Brillouin zone of graphene 2 × 2 cell. The size of orange-colored circle represents the contribution intensity of π-orbital components of graphene sheet.

The local density of states (LDOS) was calculated by partitioning the supercell into thin slabs with a thickness of $\Delta z \approx$ 0.52 Å parallel to the interface(Fig. S4). LDOS of the i-th z point $D^i(\varepsilon)$ is defined as the contribution to the total DOS from the electron density in the i-th thin slab of volume V_i:

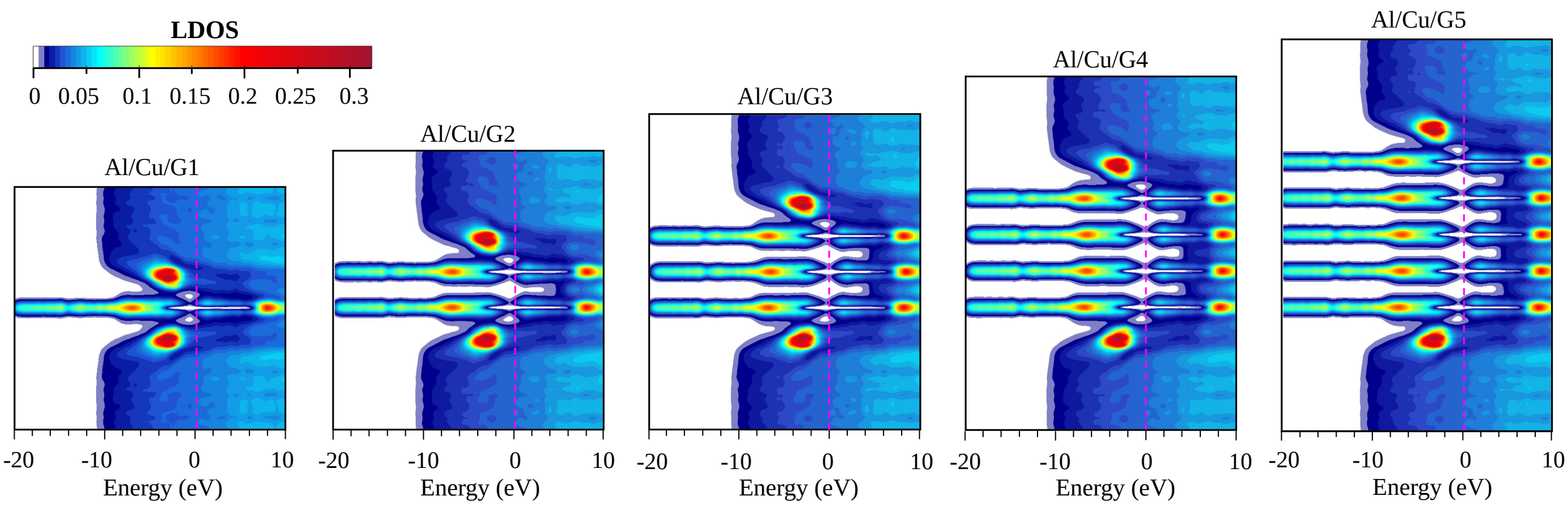

Fig. S4 LDOS of Al/Cu/Gn superlattices. The Fermi energy is set to be zero.

$$D^i(\varepsilon) = \frac{1}{V_{\mathrm{BZ}}}\sum_n \int_{V_{\mathrm{BZ}}} d\mathbf{k} W^i_{n\mathbf{k}} \delta(\varepsilon - \varepsilon_{n\mathbf{k}}) = \sum_n \sum_k w_k W^i_{nk} \delta(\varepsilon - \varepsilon_{nk}), \tag{S9}$$

where n is the band index, w_k the weight of the k-th special point, and ε_{nk} is the eigenvalue of Kohn-Sham hamiltonian. W^i_{nk} is the weight function of the i-th thin slab defined as

$$W^i_{nk} = \frac{1}{V_i}\int_{V_i} d\boldsymbol{r}|\psi_{nk}(\boldsymbol{r})|^2, \quad \text{(S10)}$$

where ψ_{nk} is the Kohn-Sham wave function. Next, the planar averaged electrostatic potential along the z-direction for Al/Cu/Gn superlattices was calculated and compared with each other(Fig. S5).

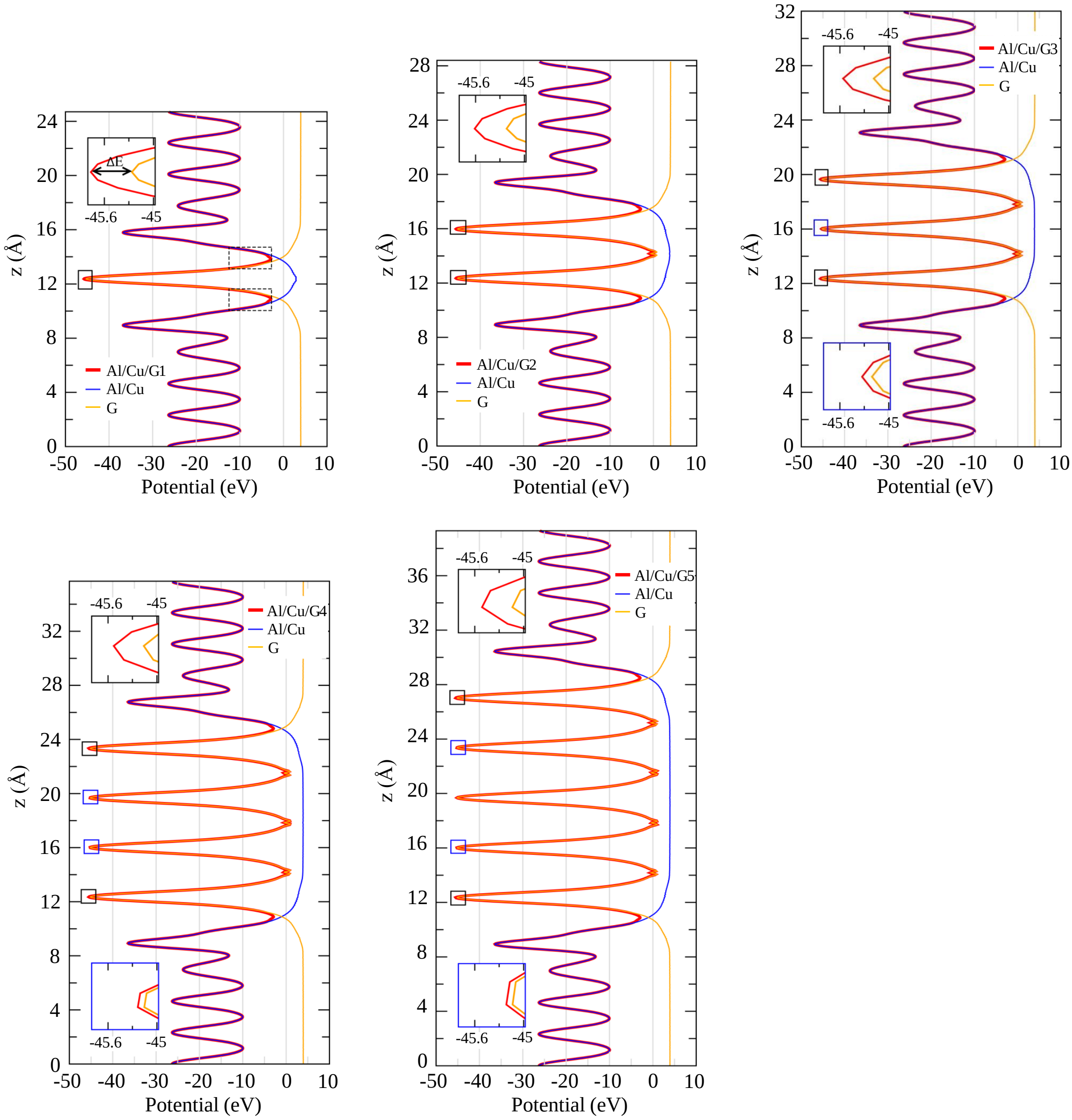

Fig. S5 Planar averaged electrostatic potential along the z direction for Al/Cu/Gn superlattices. The Fermi energy is set to be zero. Inset shows the detailed view of potential bottom.

To evaluate the relative shift of the energy level of graphene, an uniform reference vacuum energy was used in planar averaged potential lineups.

To investigate the nature of the interfacial bonding in Al/Cu/Gn composites, the integrated local density of states was calculated.

Table S5. The weight percentage of graphene (p_g), bulk modulus (B), Young's modulus (E), Poisson's ratio (ν), Pugh's ratio (μ), Dirac point position (E_D) and potential difference (ΔE) in the superlattices.(Values calculated for Al/Gn superlattices refers to our previous research [5])

n	SL	p_g (wt.%)	B (GPa)	E (GPa)	ν	μ	E_D (meV)	ΔE (meV)
1	Al/Cu/G1	8.2	101	135	0.278	1.916	−565	465
	Al/G1	14.50	93	127	0.271	1.854	−492	398
2	Al/Cu/G2	15.17	109	157	0.260	1.750	−631	394
	Al/G2	25.33	103	151	0.255	1.707	−502	278
3	Al/Cu/G3	21.15	113	268	0.251	1.675	−574	378
	Al/G3	33.72	108	166	0.247	1.642	−475	265
4	Al/Cu/G4	26.34	115	175	0.246	1.639	−530	371
	Al/G4	40.42	109	169	0.242	1.603	−440	259
5	Al/Cu/G5	30.89	117	181	0.242	1.608	−520	369
	Al/G5	45.89	113	178	0.238	1.572	−436	255

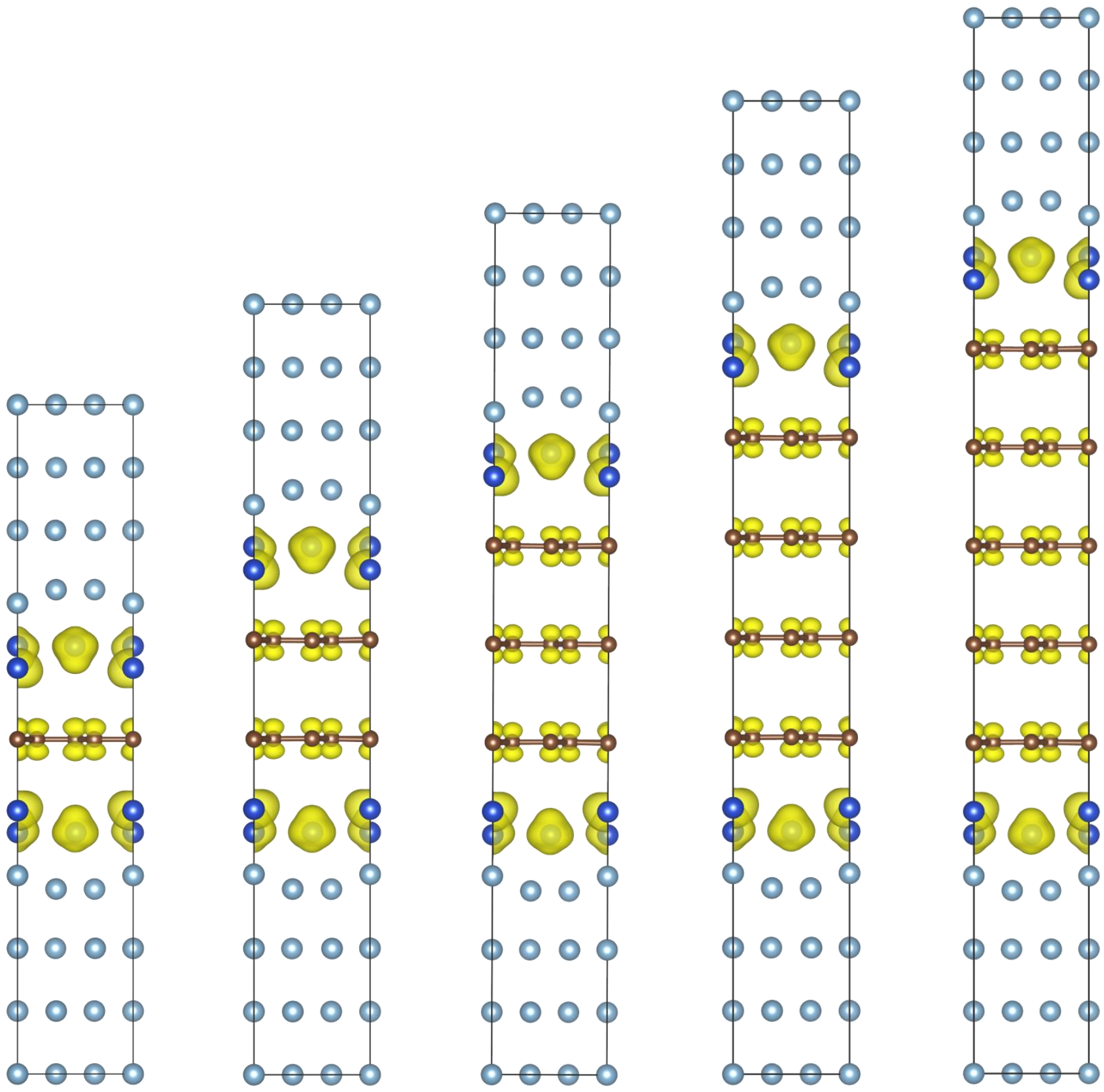

Fig. S6 Isosurface plot for integrated local density of states with energies from −3eV to −2eV.

5. Lattice Vibrational Properties

Lattice dynamics calculations were performed using the ALAMODE code [11]. The harmonic interatomic force constants (IFCs) were extracted using the $2 \times 2 \times 1$ supercell expansion and the displacement step of 0.01 Å. Meanwhile, the third- and fourth-order IFCs were computed using the $2\times2\times1$ supercell expansion. In order to calculate the harmonic phonon eigenvalues and phonon DOS, we used the second-order (harmonic) IFCs by utilizing the finite displacement method and the $2 \times 2 \times 1$ supercell, as implemented in the ALAMODE code [11]. Then, we calculated the atomic forces for each displaced configuration and extracted the harmonic IFCs by using the ALAMODE code. In order to obtain the phonon dispersions and phonon density of states (DOS) considering the temperature effect, we included the anharmonic effects at finite temperature by applying the self-consistent phonon (SCP) theory [11]. Within the SCP theory, the anharmonic phonon eigenvalues were computed as a function of temperature from the pole of the phonon Green's function beyond the perturbation theory, as implemented in the ALAMODE code. In the SCP calculations, the phonon self-energies became frequency-dependent only treating the loop diagram related with the quartic IFCs. Note that the off-diagonal elements of the self-energy should be accounted to allow the phonon eigenvectors to change by anharmonic effects. The current SCP approach applied in this work

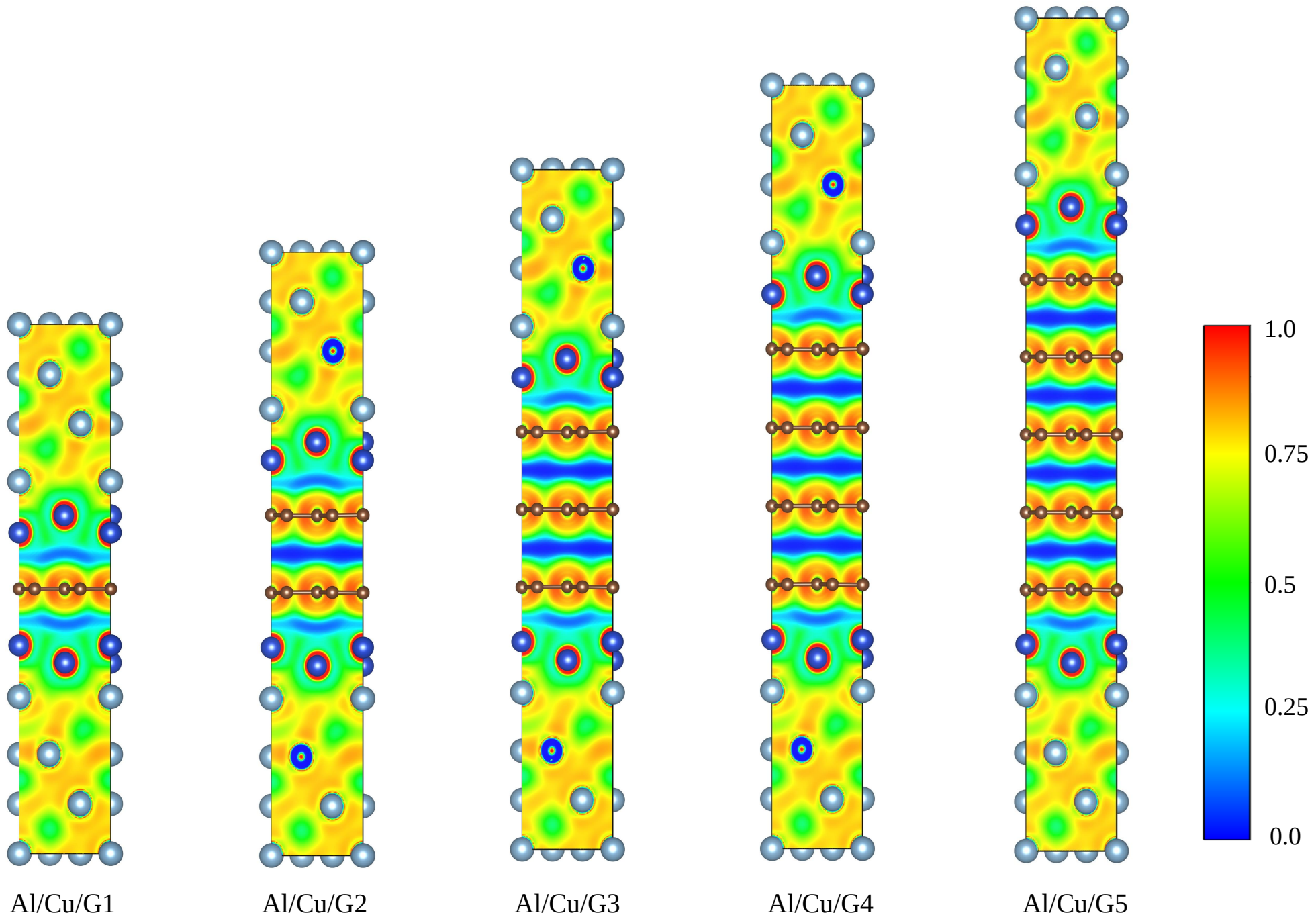

Fig. S7 Electron localization function plots in the (100) plane.

was confirmed to be efficient and accurate in accounting for severe anharmonicity in lattice dynamics and thermal transport properties of crystals [11]. To calculate the anharmonic IFCs, we employed the compressive sensing lattice dynamics (CSLD) approach [11], as implemented in the ALAMODE code. For the calculation, we prepared the 60 configurations, in which all of the atoms were displaced randomly with large displacements [11], and computed the atomic forces for each configuration by performing the DFT calculation. For the cubic and quartic IFCs, we included up to the 3rd- and 5th-nearest neighbor shells, whereas the 5th- and 6th-order IFCs were considered for the nearest-neighbor pairs. In order to estimate the accuracy of IFCs calculated by using the CSLD, we calculated the atomic forces using both the precise DFT calculations and the IFCs, providing relative errors less than 3.1% for the atomic forces.

6. Tunnelling Transmission Probability

In a one-dimensional potential field, the tunnelling transmission probability is expressed as follows.

$$D = D_0 exp(-2\int_0^L \sqrt{2m(V(x)-E)/\hbar^2}dx), \tag{S11}$$

Here, m and E are mass and energy of the electron, respectively. L is width of barrier and $V(x)$ is height of barrier. D_0 is approximately equal to 1. The potential barrier is described by quadratic function like $V(x) - E = -15.791x^2 + 25.108x$.

References

[1] P. Giannozzi, S. Baroni, N. Bonini, M. Calandra, R. Car, C. Cavazzoni, D. Ceresoli, G. L. Chiarotti, M. Cococcioni, I. Dabo *et al.* Quantum espresso: A modular and open-source software project for quantum simulations of materials. *J. Phys.: Cond. Matter*, 21:395502, 2009.

[2] Kevin F. Garrity, Joseph W. Bennet, Karin M. Rabe, and David Vanderbilt. Pseudopotentials for high-throughput dft calculations. *Comput. Mater. Sci.*, 81:446–452, 2014.

[3] J. P. Perdew, K. Burke, and M. Ernzerhof. Generalized gradient approximation made simple. *Phys. Rev. Lett.*, 77:3865, 1996.

[4] S. Grimme, J. Antony, S. Ehrlich, and H. Krieg. *J. Chem. Phys.*, 132:154104, 2010.

[5] S. J. Kim S. H. Jon J. H. Jang, K. C. Ri and C. J. Yu. Influence of interposed graphene sheets on mechanical and electronic properties of al/graphene superlattice. *Appl. Phys. Lett.*, 124:161601, 2024.

[6] D. R. Lide, editor. *CRC Handbook of Chemistry and Physics*. CRC Press, http://www.hbcpnetbase.com, Boca Raton, FL, 2005.

[7] D. R. Askeland and P. P. Fulay, editors. *Essentials of Materials Science and Engineering*. CENGAGE Learning Press, http://www.ichapters.com, 2009.

[8] T. Ohta, A. Bostwick, T. Seyller, K. Horn, and E. Rotenberg. Controlling the electronic structure of bilayer graphene. *Science*, 313:951–954, 2006.

[9] A. G. Every and A. K. McCurdy. *Table 3. Cubic system. Elements, in: Landolt-Börnstein-Group III Condensed Matter Numerical Data and Functional Relationships in Science and Technology*, volume 29a. Springer-Verlag, 1992.

[10] O. L. Blakslee, D. G. Proctor, E. J. Seldin, G. B. Spence, and T. Weng. *J. Appl. Phys.*, 41:3373, 1970.

[11] T. Tadano and S. Tsuneyuki. Quartic anharmonicity of rattlers and its effect on lattice thermal conductivity of clathrates from first principles. *Phys. Rev. Lett.*, 120:105901–105906, 2018.
